# Revolutionizing thoracic surgery education: a bibliometric analysis of the past decade’s literature

**DOI:** 10.1186/s13019-024-02947-w

**Published:** 2024-07-10

**Authors:** Chao Guo, Lei Liu, Jiaqi Zhang, Ke Zhao, Shanqing Li

**Affiliations:** Department of Thoracic Surgery, Peking Union Medical College Hospital, Shuaifuyuan 1, Wangfujing Street, Dongcheng district, Beijing, P. R. China

**Keywords:** Thoracic surgery education, Surgical skills training, Simulation-based learning, Minimally invasive surgery, Bibliometric analysis

## Abstract

**Objectives:**

Thoracic surgery is a complex field requiring advanced technical skills and critical decision-making. Surgical education must evolve to equip trainees with proficiency in new techniques and technologies.

**Methods:**

This bibliometric analysis systematically reviewed 113 articles on thoracic surgery skills training published over the past decade, retrieved from databases including Web of Science. Publication trends, citation analysis, author and journal productivity, and keyword frequencies were evaluated.

**Results:**

The United States contributed the most publications, led by pioneering institutions. Simulation training progressed from basic to sophisticated modalities and virtual reality emerged with transformative potential. Minimally invasive techniques posed unique learning challenges requiring integrated curricula.

**Conclusion:**

Ongoing investments in educational research and curriculum innovations are imperative to advance thoracic surgery training through multidisciplinary strategies. This study provides an evidentiary foundation to optimize training and address the complexities of modern thoracic surgery.

## Introduction

Thoracic surgery is an important branch of surgical science. Compared to other surgical specialties, thoracic surgery procedures are more challenging, the disease spectrum is more complex, and patients often present with more critical conditions [1]. In addition, thoracic surgeons face pressures from multiple fronts, including demands for mastery of surgical skills to ensure patient safety, extensive knowledge base, and timely and accurate evaluation of medical conditions. The high stakes nature of thoracic surgery procedures also contributes to the stress [2]. 

Surgical education in thoracic surgery has always occupied an indispensable position in the training of healthcare professionals. With continuous advancements and innovations in medical technology, the field of thoracic surgery has undergone a significant transformation from traditional open surgeries to minimally invasive procedures [3]. This shift necessitates that thoracic surgeons acquire a range of new skills and knowledge. Consequently, the methods and content of thoracic surgical education must evolve to meet the demands of a modern healthcare system.

In such an advancing field, bibliometric analysis has emerged as an essential tool for evaluating trends and focal points in the published literature pertaining to thoracic surgical skills education. It provides insights into current research hot spots and gaps through the quantification of scientific publications in the field, offering data support for future research directions and education system reforms. In this article, we will conduct a bibliometric analysis of the trends and developments in the field of thoracic surgical skills education over the past decade, aiming to provide evidence and direction for future innovations and curriculum development.

## Materials and methods

This bibliometric study aims to analyze and quantify the scientific literature on thoracic surgical skills education over the past decade. We employed a web-based literature retrieval approach, utilizing the Web of Science Core Collection databases, renowned for their comprehensive citation search capabilities and indexing of peer-reviewed high-impact journals.

### Data collection

The search strategy focused on capturing a broad range of publications related to thoracic surgery education, surgical skills training, simulation, minimally invasive surgical techniques, and related technological advancements. We included the following thematic categories: “thoracic surgery skills instruction”, “thoracic surgical dexterity tutoring”, “thoracic surgery techniques coaching”, “thoracic surgery skill teaching” and “thoracic surgery simulation-based trainings”.

We limited our search to articles published in the English language between January 1, 2014, and December 15, 2023. Original research articles, reviews and editorials were considered for inclusion. Exclusion criteria entailed non-English articles, articles outside the selected date range, and conference abstracts unless they presented novel research findings.

### Search strategy

An advanced search query was constructed using a combination of Boolean operators (AND, OR, NOT) to refine the search results. Keywords related to the thematic categories were included in the search query, and Medical Subject Headings (MeSH) terms were utilized where applicable.

### Data analysis

Upon the retrieval of articles, we exported data for bibliometric analysis, which included information on publication year, authors, institutions, countries, citation counts, and keywords. The citation analysis was used to evaluate the impact of publications on the scientific community. Co-authorship and co-occurrence of keywords were analyzed to identify research trends, collaboration patterns, and hotspots within the field of thoracic surgical skills education.

### Statistical and bibliometric tools

We utilized specialized bibliometric software and statistical tools (VOSviewer) for data visualization, to create network maps illustrating the connections between organizations, keywords, and authors. VOSviewer also facilitated the assessment of the most influential articles, journals, and researchers within the field.

Ethical approval or patient consent was not required for this study, as our analysis was based on publicly available data and did not involve human participants, medical records, or animal subjects.

## Results

The number of included articles on thoracic surgical skills education published in the past decade was 113. The number of citations for these articles ranged from 0 to 69, with a total of 1,206 citations as of December 11, 2023. Three articles were cited more than 50 times, and seven articles were cited over 30 times each. The year with the highest number of published articles was 2018, with 19 articles. Annals of Thoracic Surgery accounted for the largest share of included articles, with 27. According to the latest 2022 Impact Factors released in 2023, the top 3 journals were Annals of Thoracic Surgery, European Journal of Cardio-Thoracic Surgery, and Surgical Endoscopy and Other Interventional Techniques (Table 1). The included articles consisted of 86 original articles, 6 reviews, and 21 editorial materials.

**Table 1:** Journals and their impact factors publishing more than 5 articles in the included articles on thoracic surgery education

Rank	Journal	Articles	Citations	Impact Factor
1	Annals of Thoracic Surgery	27	351	4.6
2	Journal of Thoracic and Cardiovascular Surgery	12	256	6
3	Journal of Thoracic Disease	12	59	2.5
4	Interactive Cardiovascular and Thoracic Surgery	8	79	1.7
5	Thoracic Surgery Clinics	8	41	2.1
6	Video-Assisted Thoracic Surgery	7	10	0.2
7	Surgical Endoscopy and Other Interventional Techniques	6	145	3.1
8	European Journal of Cardio-Thoracic Surgery	5	44	3.4

**Fig. 1:**
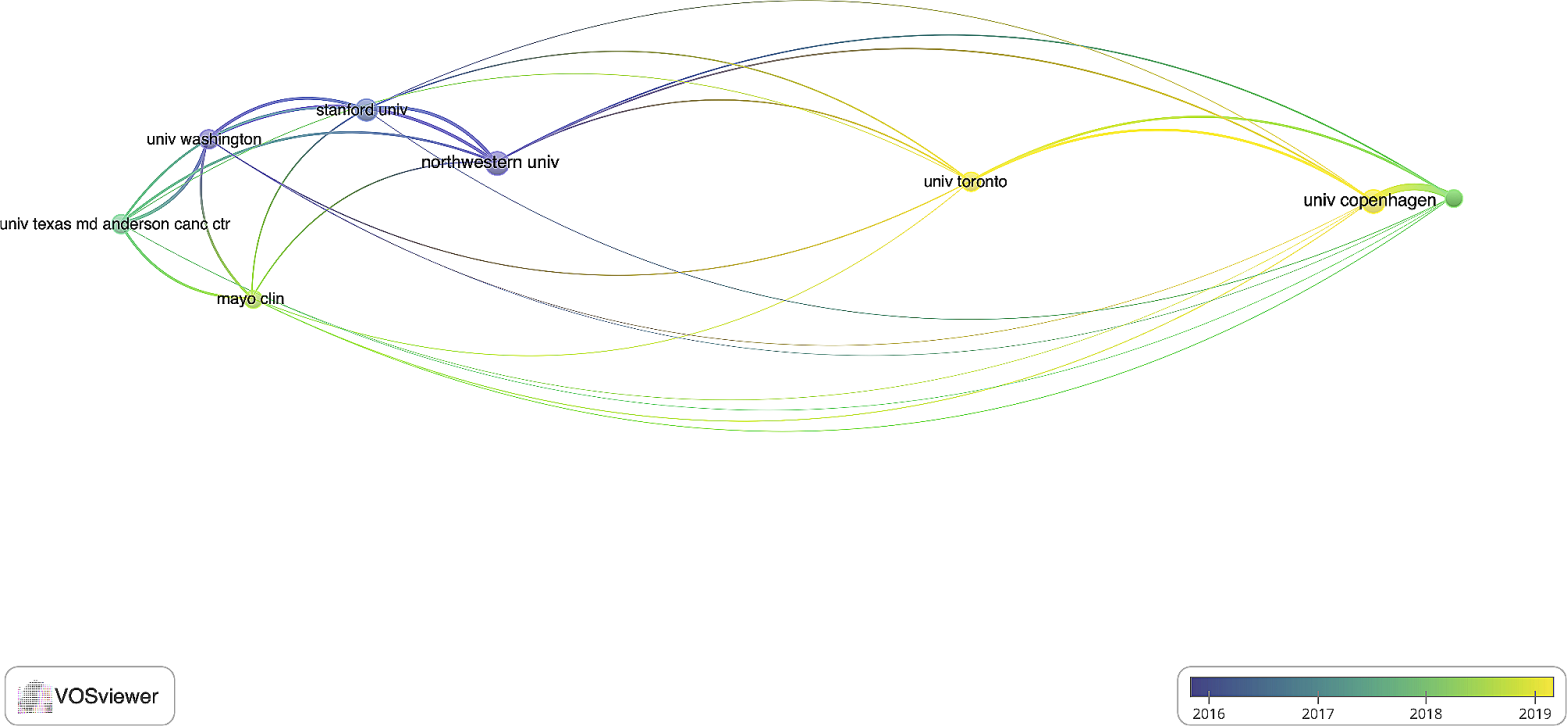
Coupling analysis of organizations publishing more than 5 articles in the included articles on thoracic surgery education: blue: earlier, yellow: later;

**Fig. 2:**
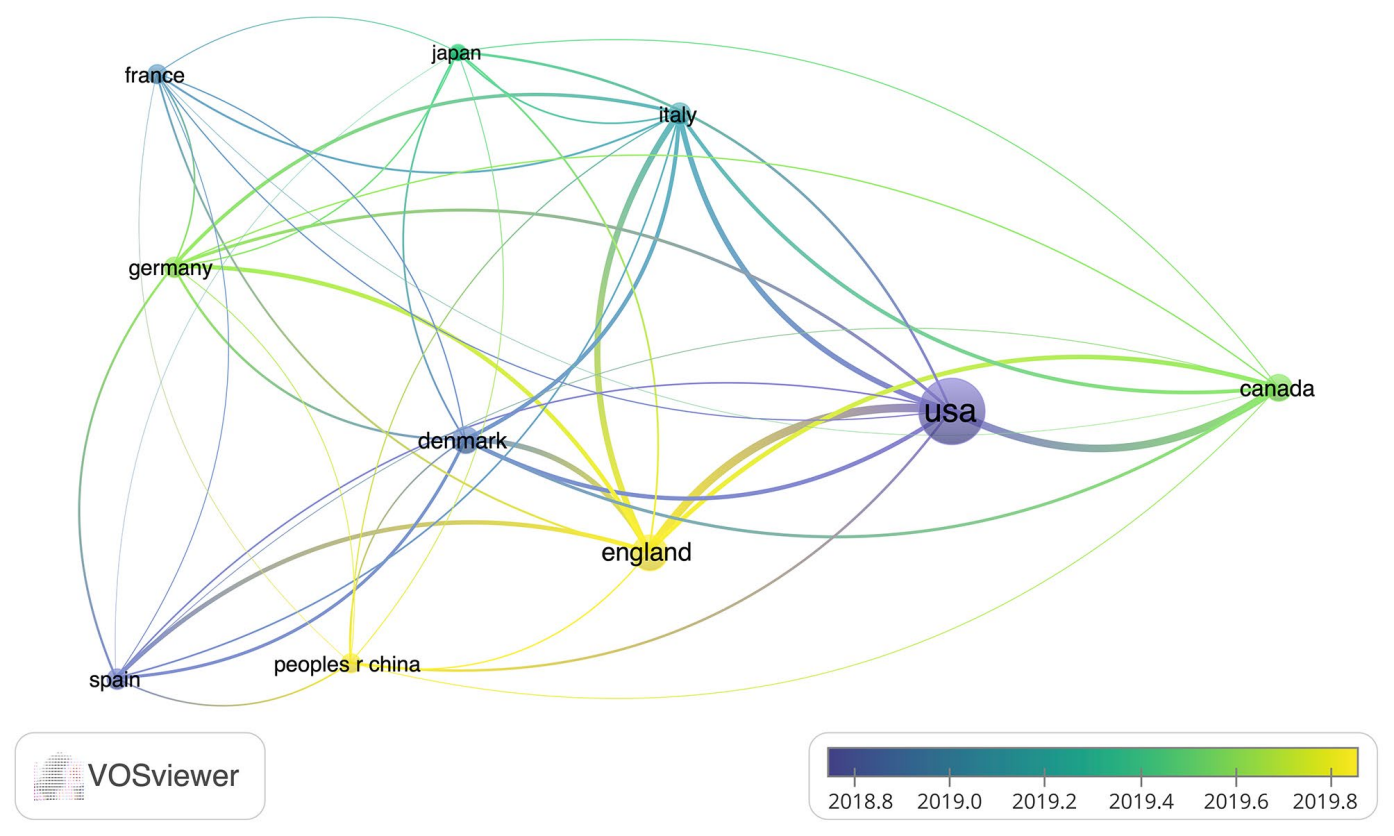
Coupling analysis of countries publishing more than 5 articles in the included articles on thoracic surgery education: blue: earlier, yellow: later

**Fig. 3:**
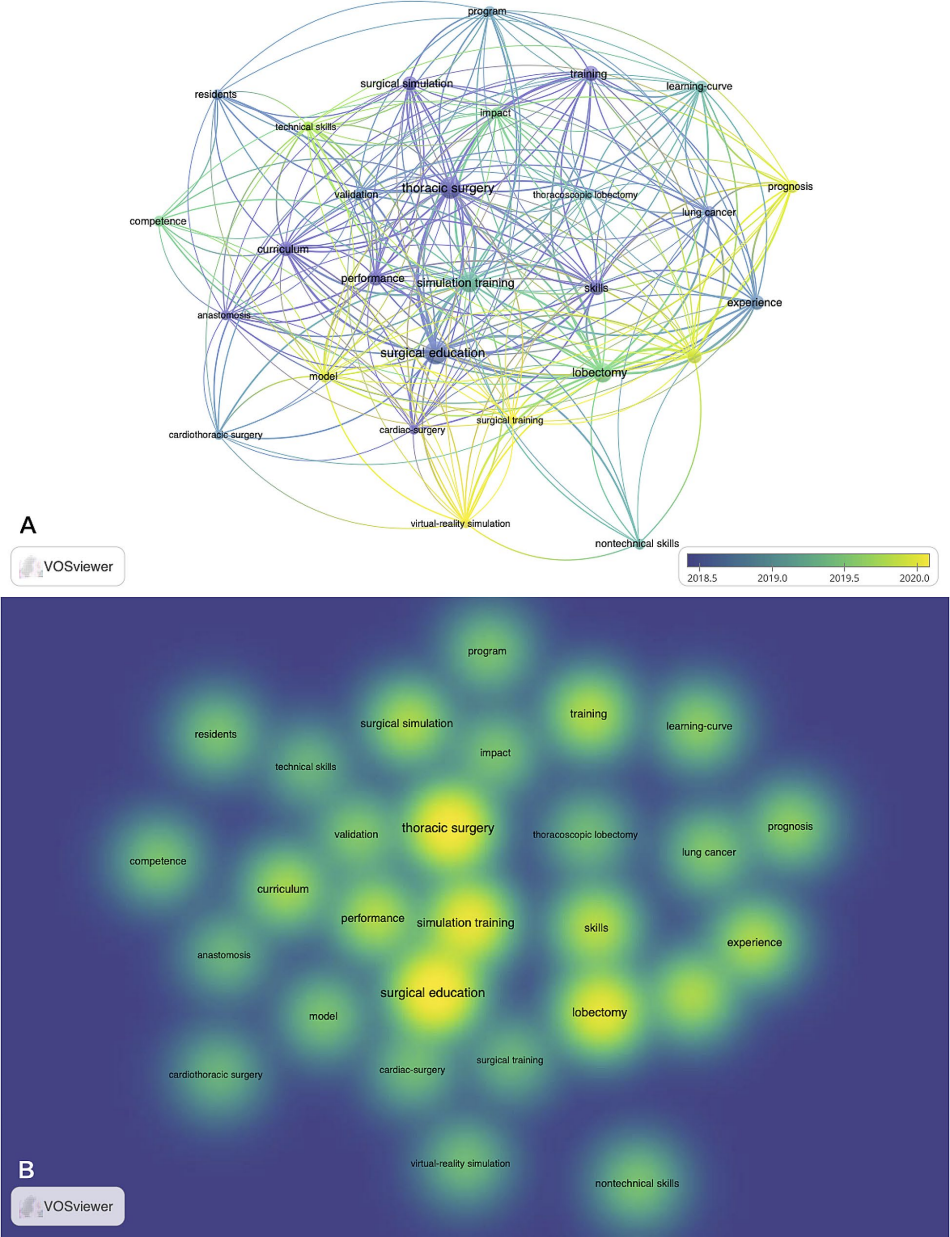
Co-occurrence analysis of keywords. (**A**) Distribution of keywords according to average publication year (blue: earlier, yellow: later). (**B**) Distribution of keywords according to the mean frequency of appearance. Keywords in yellow occurred with the highest frequency

Among the authors of the included works, Konge Lars, Petersen Rene Horsleben Hansen, and Henrik Jessen had the top 3 number of articles and citations, with 234, 223, and 216 citations and 11, 9, and 8 articles, respectively (Table 2). The included articles came from 265 organizations, with the top 3 being Northwestern University, University of Copenhagen, and Stanford University (Table 3; Fig. 1). The articles originated from 32 countries, with the USA, Denmark, and England producing the top 3 most cited papers (Table 4; Fig. 2). Of the included articles in this study, the top 5 keywords were surgical education, thoracic surgery, simulation training, lobectomy, and skill (Fig. 3).

**Table 2:** Authors that contributed 5 or more articles in included articles on thoracic surgery education

Rank	Author	Article	Citations
1	Konge Lars	11	234
2	Petersen Rene Horsleben	9	223
3	Hansen Henrik Jessen	8	216
4	Jensen Katrine	6	201
5	Bjerrum Flemming	5	108
6	Massard Gilbert	5	26
7	Vaporciyan Ara A.	5	125
8	Verrier Edward D.	5	140

**Table 3:** Organizations of origin publishing more than 5 articles in the included articles on thoracic surgery education

Rank	Organization	Article	Citations
1	Northwestern University	8	191
2	University of Copenhagen	8	171
3	Stanford University	7	160
4	University of Washington	6	158
5	Rigshospitalet	5	148
6	The University of Texas MD Anderson Cancer Center	6	127
7	University of Toronto	6	90
8	Mayo Clinic	5	64

**Table 4:** Countries publishing more than 5 articles in the included articles on thoracic surgery education:

Rank	Country	Article	Citations
1	U.S.A	53	644
2	England	18	148
3	Denmark	11	234
4	Canada	11	124
5	Italy	8	64
6	Spain	7	58
7	Germany	7	49
8	France	6	47
9	China	6	44
10	Japan	5	32

## Discussion

The perusal of thoracic surgical skills training literature from the past decade through a bibliometric lens paints a multifaceted picture of the current state and evolution of the field. The following discussion provides a deeper dissection of the trends and developments in four key areas.

An in-depth appraisal of the literature underscores the United States’ commanding role in publishing on thoracic surgical education, a phenomenon that can be dissected into several underlying factors. Institutions in the U.S. have historically pioneered numerous surgical techniques and educational methods, supporting a legacy of excellence and innovation in surgical training [4, 5]. The Accreditation Council for Graduate Medical Education (ACGME) and other regulatory bodies have progressively embraced competency-based education frameworks, exerting global influence on training standards [6, 7]. Moreover, the integration of multidisciplinary approaches involving educational psychologists, learning technologists, and seasoned surgeons has fostered a fertile environment for research activity and pedagogical advancement in the U.S. [8, 9]. This concerted approach has led to a research output that not only quantifies but also qualitatively enriches the field, serving as a beacon for international thoracic surgical education practices.

Simulation in thoracic surgery education has evolved from a nascent adjunct to a pivotal component in surgical training [2, 10]. The construct validity of simulators has been scrutinized and significantly improved over time, enabling a more accurate replication of the haptic and visual feedback experienced during actual surgery [11]. The transition from low-fidelity to high-fidelity models and the advent of patient-specific simulation tailored to individual anatomy further underscore the technological strides made [12]. Progressive curricular integration of simulation-based training is shown to enhance not only technical skills but also non-technical attributes, such as teamwork, communication, and crisis management—crucial elements for the high-stakes realm of thoracic surgery [13]. 

Virtual reality (VR) has arguably brought about a paradigm shift in how surgical training can be conceptualized and delivered. Early concerns over VR’s utility and cost-efficiency have progressively given way to evidence demonstrating its efficacy in augmenting complex cognitive skill sets through immersive, repeated, and deliberate practice [14]. Recent studies have emphasized the utility of VR in facilitating a three-dimensional understanding of thoracic anatomy, an aspect critical in navigating the confined spaces of thoracic cavities [15]. Additionally, VR’s burgeoning role in remote and distributed learning is particularly pertinent in the context of the recent global challenges posed by the COVID-19 pandemic, allowing trainees to transcend geographical barriers and maintain skill acquisition in socially distanced settings [16, 17]. 

The growing preference for minimally invasive techniques, including VATS and RATS, presents unique educational challenges characterized by a steep learning curve. These modalities require completely different skillsets: while VATS emphasizes leveraging endoscopic visuals and refining hand-eye coordination, RATS demands dexterity in manipulating robotic arms and mastering console-based interfaces [18, 19]. Mastery in these areas necessitates an integrated training approach comprising didactic components, dry laboratory practice, hands-on sessions with simulators or cadaveric tissue, and mentored clinical immersion. Through a blend of simulation, mentorship, and graduated responsibility, training paradigms can efficiently and effectively prepare surgeons to meet and surpass the high-quality benchmarks established in thoracic surgery [20]. 

In light of the evolving landscape of thoracic surgery education, our study’s findings underscore the necessity for integrating advanced technologies such as simulation-based training and VR into the curricular framework. This integration calls for a paradigm shift towards competency-based, modular curricula that cater to the varied learning requirements of surgical trainees, emphasizing both technical proficiency and the development of essential non-technical skills. Recognizing the pivotal role of educational research in this progression, we advocate for the establishment of interdisciplinary research centers. These centers would serve as collaborative platforms for surgeons, educators, technologists, and educational psychologists, driving innovation in pedagogical approaches and educational technology. To operationalize this vision, we propose strategies including the creation of immersive learning modules, the adoption of a structured competency-based progression system, and the promotion of interprofessional education to enhance collaborative practice. Additionally, we highlight the importance of remote learning opportunities and real-time assessment tools to facilitate continuous improvement. Looking ahead, we identify key areas for future research, such as longitudinal studies to evaluate the long-term impact of technology-integrated curricula on patient outcomes, comparative effectiveness research on educational modalities, and the exploration of adaptive learning systems. The ethical integration of advanced technologies and the promotion of global collaboration to establish standardized educational protocols are also critical. By embracing these strategies and research avenues, the field of thoracic surgery education can continue to innovate, ensuring that training programs are equipped to meet the challenges of modern healthcare and ultimately enhance patient care.

This bibliometric analysis, while providing a valuable foundation for understanding the landscape of thoracic surgery education literature, acknowledges several inherent limitations. The focus on English publications and a select set of databases may have led to the exclusion of pertinent literature, and the omission of conference abstracts might have overlooked early research insights. We recognize that citation analysis metrics, which can favor longevity over quality, do not fully account for the rigor and impact of the studies. Additionally, publication and citation rates might be influenced by factors such as journal prestige, rather than solely reflecting research merit. The cross-sectional design of our study offers a snapshot of the current state of research but does not capture its longitudinal evolution. Furthermore, our quantitative approach, while comprehensive, does not delve into the qualitative aspects of the studies, such as their methodological rigor and the significance of their findings. Despite these limitations related to literature search scope, analytic metrics, dataset coverage, and study design, our analysis serves as a substantial evidentiary base that can inform and guide future advancements in thoracic surgery education, and also underscores the need for qualitative research to complement these findings.

## Conclusion

In conclusion, our study advocates for the integration of cutting-edge technologies like simulation and VR into thoracic surgery education, necessitating a shift towards competency-based, modular curricula. This approach is designed to accommodate the diverse learning needs of trainees and enhance both technical and non-technical skills. We emphasize the establishment of interdisciplinary research centers to innovate pedagogical methods and educational technology, and propose strategies that include immersive learning, structured progression, and interprofessional collaboration. Recognizing the importance of continuous improvement, we also stress the need for future research to assess the long-term impact of these educational advancements on patient outcomes. As we look to the future, sustained investment in educational research and curriculum innovation is key to maintaining the momentum of progress and ensuring that thoracic surgery training remains at the forefront of medical education, ultimately improving patient care.

## Data Availability

No datasets were generated or analysed during the current study.

## Abbreviations

MeSH: Medical Subject Headings
ACGME: Accreditation Council for Graduate Medical Education
VR: Virtual reality
